# Anti-Tumor Effects of *Ganoderma lucidum* (Reishi) in Inflammatory Breast Cancer in *In Vivo* and *In Vitro* Models

**DOI:** 10.1371/journal.pone.0057431

**Published:** 2013-02-28

**Authors:** Ivette J. Suarez-Arroyo, Raysa Rosario-Acevedo, Alexandra Aguilar-Perez, Pedro L. Clemente, Luis A. Cubano, Juan Serrano, Robert J. Schneider, Michelle M. Martínez-Montemayor

**Affiliations:** 1 Department of Biochemistry, Universidad Central del Caribe, School of Medicine, Bayamón, Puerto Rico, United States of America; 2 Department of Anatomy and Cell Biology, Universidad Central del Caribe, School of Medicine, Bayamón, Puerto Rico, United States of America; 3 San Pablo Pathology, Bayamón, Puerto Rico, United States of America; 4 New York University Cancer Institute, New York, New York, United States of America; University of Pecs Medical School, Hungary

## Abstract

The medicinal mushroom *Ganoderma lucidum* (Reishi) was tested as a potential therapeutic for Inflammatory Breast Cancer (IBC) using *in vivo* and *in vitro* IBC models. IBC is a lethal and aggressive form of breast cancer that manifests itself without a typical tumor mass. Studies show that IBC tissue biopsies overexpress E-cadherin and the eukaryotic initiation factor 4GI (eIF4GI), two proteins that are partially responsible for the unique pathological properties of this disease. IBC is treated with a multimodal approach that includes non-targeted systemic chemotherapy, surgery, and radiation. Because of its non-toxic and selective anti-cancer activity, medicinal mushroom extracts have received attention for their use in cancer therapy. Our previous studies demonstrate these selective anti-cancer effects of Reishi, where IBC cell viability and invasion, as well as the expression of key IBC molecules, including eIF4G is compromised. Thus, herein we define the mechanistic effects of Reishi focusing on the phosphoinositide-3-kinase (PI3K)/AKT/mammalian target of rapamycin (mTOR) pathway, a regulator of cell survival and growth. The present study demonstrates that Reishi treated IBC SUM-149 cells have reduced expression of mTOR downstream effectors at early treatment times, as we observe reduced eIF4G levels coupled with increased levels of eIF4E bound to 4E-BP, with consequential protein synthesis reduction. Severe combined immunodeficient mice injected with IBC cells treated with Reishi for 13 weeks show reduced tumor growth and weight by ∼50%, and Reishi treated tumors showed reduced expression of E-cadherin, mTOR, eIF4G, and p70S6K, and activity of extracellular regulated kinase (ERK1/2). Our results provide evidence that Reishi suppresses protein synthesis and tumor growth by affecting survival and proliferative signaling pathways that act on translation, suggesting that Reishi is a potential natural therapeutic for breast and other cancers.

## Introduction

Inflammatory breast cancer (IBC) is a rare, aggressive and lethal type of breast cancer that particularly involves hyper-activation of protein synthesis pathways. In IBC, cancer cells block dermal lymphatics of the breast causing an inflammatory phenotype. IBC lethality derives from generation of tumor emboli, which are non-adherent cell clusters that rapidly spread into the dermal lymphatics by a form of continuous invasion known as passive metastasis. Despite improvements in survival and outcomes for breast cancer generally over the last 20 years, patients with IBC continue to have a poorer prognosis with 5-year survival rates of 50% [1], whereas the average comparable rates for patients with non-inflammatory breast cancers are 70% to 80%. Standard IBC treatment involves non-targeted chemotherapy or a combination of several agents including radiation therapy, hormonal therapy and surgery. The systemic treatment utilized to treat IBC causes generalized destructive effects affecting both cancerous and non-cancerous cells, thus new therapeutic strategies are highly desirable to improve the prognoses of women with inflammatory carcinoma.

*Ganoderma lucidum*, also known as Reishi, is a traditional Chinese medicinal mushroom that has been used for centuries in East Asia to treat a variety of diseases, such as immunological disorders, inflammation and cancer [2]. The effectiveness of Reishi has been attributed to either the polysaccharide fraction, which is responsible for the stimulation of the immune system, or to the triterpenes, which demonstrate cytotoxic activity against a variety of cancer cells [3], [4], [5]. *G. lucidum* extract (GLE), containing polysaccharides and triterpenes, was reported to suppress growth and metastatic potential of human MDA-MD-231 breast cancer cells by inhibiting the activity of Akt and transcription factors AP-1 and NF-κB, resulting in the downregulation of expression of cyclin D1 [6], [7], [8]. Moreover, we recently reported that Reishi selectively inhibits SUM-149 IBC cell viability and invasion, while not affecting non-cancerous mammary epithelial (MCF10A) cell viability, making it a potential anti-cancer therapeutic [9].

Deregulation of phosphoinositide 3-kinase (PI3K)/AKT/mammalian target of rapamycin (mTOR) pathway, and mRNA translation from negative feedback responses, is associated with increased transformation and oncogenesis [10]. More than 70% of breast tumors have molecular alterations in at least one component of the pathway [11]. Loss of *PTEN*, which is a tumor-suppressor gene that inhibits the PI3K/AKT/mTOR pathway, causes the aberrant mTOR pathway activation. Activation of mTOR signaling is involved in some of the cancer hallmarks and contributes to tumor growth, angiogenesis and metastasis, reinforcing the importance of considering mTOR targeting in cancer therapy [12], [13], [14]. mTOR forms two functionally distinct multiprotein complexes, mTORC1 and mTORC2, that control cell growth, proliferation, and survival. mTORC1 effectors are the eukaryotic initiation factor 4E-binding protein 1 (4E-BP1) and the ribosomal protein S6 kinase (p70S6K) [15], [16]. mTORC1 acts on the cap-dependent translation initiation complex known as eIF4F, consisting of cap binding protein eIF4E, RNA helicase eIF4A and molecular scaffold protein eIF4G. Directed by mTORC1, the 4E-BP-eIF4E complex exerts control over cap-dependent translation, assisted by p70S6K, which also stimulates eIF4F translation of specific mRNAs with highly structured 5′UTRs via eIF4B phosphorylation, promoting cell growth, and tumor progression [16]. IBC tissues overexpress the eukaryotic initiation factor 4G (eIF4G), and this effect is usually seen in the absence of any changes in the levels of eIF4E [17]. In addition, in SUM-149 IBC cells, a substantial amount of eIF4G is free and unbound to eIF4E, because IBC cells have a high proportion of their 4E binding protein (4E-BP1) in the hypophosphorylated (activated) state and bound to eIF4E, compared with MCF10A cells that have similar levels of eIF4G and total 4E-BP1 [17]. Overexpression of eIF4G drives greater formation of eIF4F and cap-dependent mRNA translation, and high levels of eIF4G maintain high levels of internal ribosome entry sites (IRES)-dependent (eIF4E-independent) mRNA translation as well, which is used as an alternative mechanism of translation initiation by subset of stress response, and survival mRNAs. Importantly, eIF4G is responsible for the strong homotypic IBC cell interaction that drives formation of tumor emboli (tumor spheroids *in vitro*) and promotes IBC cell invasion and passive metastasis [17].

The present study was designed to elucidate the anti-tumor effects of Reishi utilizing in vivo and *in vitro* IBC models focusing on the PI3K/AKT/mTOR pathways and effectors. We tested our hypothesis that Reishi extract acts on mTORC1 and/or downstream effector proteins by using an IBC model that depends on this pathway. Our findings are the first to show that Reishi downregulates the expression of PI3K/AKT/mTOR and also MAPK pathway effector genes and proteins *in vitro* and *in vivo*, and it significantly reduces IBC tumor growth and weight. The current results suggest that Reishi is a potential novel chemoprevention and treatment agent for IBC and other malignancies that are mTOR driven.

## Materials and Methods

### Ethics Statement

All animal studies were conducted under approved protocol #11-XVI-01 by the Universidad Central del Caribe School of Medicine, Institutional Animal Care and Use Committee in accordance with the principles and procedures outlined in the *NIH Guideline for the Care and Use of Laboratory Animals*.

### Cell Culture

The patient derived IBC cell line SUM-149 (from Dr. Steven Eithier, University of Michigan, Ann Arbor, MI) [18] was cultured in Ham’s F12 medium (Life Technologies, Carlsbad, CA) with 10% fetal bovine serum (FBS) as in [9]. Human mammary epithelial MCF10A cells (from ATCC, Manassas, VA) were cultured in Dulbecco’s modified Eagle’s medium (DMEM) and Ham’s F12 medium (Life Technologies) with 10% horse serum (Sigma Aldrich, St. Louis, MO).

### Whole Mushroom Reishi Extract

A commercially available extract consisting of Reishi fruiting body and cracked spores, known as ReishiMax GLp™, was purchased from Pharmanex® Inc. (Provo, UT). Details on the preparation of this extract are described in [19]. The extract is available in capsules, where the contents (500 mg) were dissolved in 100% sterile Dimethyl Sulfoxide (DMSO, Sigma Aldrich) at a working stock of 50 mg/mL, then diluted to 0.5 mg/mL with media before use. This concentration was chosen after a thorough review of the literature, and it is the concentration we demonstrate to be effective for anti-IBC effects [9]. For in vivo studies, Reishi extract was dissolved in the vehicle solution of 10% ethanol +90% ultrapure water at a concentration of 28 mg/kg BW.

### In vivo Study

Female severe combined immunodeficient (SCID) mice (21 d of age) were purchased from Charles River Laboratories International Inc. (Wilmington, MA) and housed under specific pathogen-free conditions. The mice received autoclaved AIN 76-A phytoestrogen-free diet (Tek Global, Harlan Teklad, Madison, WI) and water ad libitum. Cell inoculations were performed as previously described by us [20]. SUM-149 cells (∼1×10^6^) in Matrigel (BD Biosciences, San Jose, CA) were injected into the mammary fat pad under isofluorane inhalation as described in [20], [21]. After tumor establishment (1 week post-inoculation), the animals were randomly divided into control (n = 11) and experimental (n = 11) groups. Mice were gavaged every day with vehicle or 28 mg/kg BW of Reishi for a period of 13 week. Mice were weighed weekly and tumor volume was measured once a week along two major axes using calipers measurements. Tumor volume (mm^3^) was calculated as follows: π/6 (L)(W)(H). The relative tumor volumes were calculated as the ratio of the average tumor volume on week n divided by the average tumor volume on week one. Tumor weights were obtained at the end of the study.

### Real Time RT-PCR Analysis

Gene expression profiles were obtained from SUM-149 cells treated with 0 or 0.5 mg/mL Reishi for 3 hours and from 30 mg of tumors extracted from mice gavaged with 0 or 28 mg/kg BW Reishi. Total RNA extraction and gDNA elimination was performed using the Qiagen RNeasy Kit (Qiagen, Valencia, CA). RNA concentration was detected using a NanoDrop (NanoDrop Technologies, Wilmington, DE). RNA (500 ng) was used to synthesize cDNA using C-03 RT^2^ First Strand Kit (SA Biosciences, Frederick, MD), and gene expression profiles of 84 genes were investigated using the human PI3K/AKT/mTOR Signaling Pathway (PAHS-058A) RT^2^ Profiler™ PCR arrays (SA Biosciences, Frederick, MD). Gene expression levels were individually assessed using the 2^(−ΔCt)^ formula by comparing the relative gene expression of 84 genes to reference genes and reproducibility was maintained by using three biological replicates from three individual experiments or three different tumors from vehicle or Reishi treated mice. Cell cycle regulatory gene expression profiles were obtained from SUM-149 cells treated with 0 or 0.5 mg/mL Reishi for 3, 6, 24 or 48 hours. The real time RT-PCR primers sequences, gene names and the websites used for their design are summarized in **Table 1**. Real time reactions were performed using SsoAdvanced SYBR Green Supermix (BioRad) in a 20µL reaction. The analyses were conducted using triplicate cDNA samples from three individual experiments.

**Table 1:** A list of the genes and primers used in the Real Time RT-PCR analysis.

Gene	Primer sequence
*CCNA2*	Forward: 5′-GCTGGAGCTGCCTTTCATTTAGCA-3′
	Reverse: 3′-ATGCTGTGGTGCTTTGAGGTAGGT-5′
*CCNB2*	Forward: 5′-AAAGCTCAGAACACCAAAGTTCCA-3′
	Reverse: 3′-ACAGAAGCAGTAGGTTTCAGTTGT-5′
*CCND1*	Forward: 5′-TGGTGAACAAGCTCAAGTGGAACC-3′
	Reverse: 3′-TGATCTGTTTGTTCTCCTCCGCCT-5′
*WEE1*	Forward: 5′-ATTCAGTATTGCTGTCCGCTTCTA-3′
	Reverse: 3′-TTTGCCATCTGTGCTTTCTTGA-5′
*RPL13A*	Forward: 5′-TGAAGCCTACAAGAAAGTTTGCCT-3′
	Reverse: 3′-TAGCCTCATGAGCTGTTTCTTCTT-3′

Real time PCR primers were designed using the websites: www.idtdna.com, www.basic.northwestern.edu/biotools/oligocalc.html, http://blast.ncbi.nlm.nih.gov/Blast. cgi, and synthesized at Sigma-Genosys (St. Louis, MI).

### Western Blot Analysis

SUM-149 cells treated with 0 or 0.5 mg/mL Reishi for 2, 4 or 6 hours were lysed and western blotted as previously described by us [9]. We used a monoclonal anti-Phospho-mTOR (Ser2481), monoclonal anti-mTOR, anti-Phospho-4E-BP1 (Thr37/46), anti-Phospho-S6 Ribosomal Protein (Ser235/236), anti-S6 Ribosomal Protein, anti-Akt, anti-Phospho-Akt (Ser473), anti-eIF4G and anti-eIF4E and polyclonal anti-p70s6 kinase (Cell Signaling, Danvers, MA) antibodies. Flash frozen primary tumors (30 mg) were lysed using a homogenizer (Brinkmann Polytron, Mississauga, ONT, Canada) in lysis buffer (10% SDS, 10% sodium deoxycholate, 1% Triton-X 100, 1% Igepal, and protease and phosphatase inhibitors and quantified using the Precision Red protein assay kit (Cytoskeleton, Inc. Denver, CO) as previously described by us [20]. Equal total protein amounts were resolved on SDS-PAGE gels and Western blotted using monoclonal anti-E-cadherin (Santa Cruz Biotechnology, Santa Cruz, CA), anti-p120 catenin (Epitomics, Burlingame, CA), anti-Phospho-mTOR (Ser2448), polyclonal anti-c-Myc (Cell Signaling) and for proteins described above.

### Metabolic Labeling

Protein synthesis assays were conducted as described in [17]. Briefly, cells (1×10^5^) treated with 0 mg/mL (n = 3/vehicle) or 0.5 mg/mL Reishi (n = 3/treatment) for 6 and 24 hours were incubated with 20µCi of 35S-methionine/cysteine per ml (Easytag Express protein labeling mix; Dupont/NEN) in methionine/cysteine - free DMEM for 1 hour, washed twice with ice-cold phosphate-buffered saline (PBS) and lysed by incubation in 0.5% NP-40 lysis buffer (0.5% NP-40, 50 mM HEPES, pH 7.0, 250 mM NaCl, 2 mM EDTA, 2 mM sodium orthovanadate, 25 mM glycerophosphate and complete protease inhibitor (Roche) for 10 min at 4°C. Lysates were clarified by centrifugation at 13,000 RPM for 10 min at 4°C. Specific activity of methionine incorporation was determined by trichloroacetic acid precipitation onto GF/C filters and liquid scintillation counting. Assays were conducted in duplicate and repeated at least three times.

### Cap Binding Assay

SUM-149 cells were treated with 0 mg/mL (n = 3/vehicle) or 0.5 mg/mL Reishi (n = 3/treatment) for 4, 6 and 24 hours. Cells were lysed on ice and total proteins were extracted using NP-40 lysis buffer. Protein concentrations were determined with Precision Red™ advanced protein measurement reagent (Cytoskeleton, Denver, CO) according to the manufacturer’s instructions. For cap-affinity chromatography, 250µg of lysate were combined with 30µL of 7-methyl GTP-Sepharose (immobilized cap-analog, GE Healthcare) as described in [22]. Affinity purifications were performed overnight at 4°C with rotation. After washing the resin three times with NP-40 lysis buffer, bound proteins were eluted with 6X SDS sample buffer. The eluted proteins were visualized by performing Western blot with anti-eIF4G (Cell Signaling), eIF4E (BD Transduction Laboratories), anti-eIF4A (gift of W. Merrick, Case Western Reserve University, Cleveland, OH), anti-4E-BP1 (Cell Signaling) and β-tubulin (BD Pharmigen, Franklin Lakes, NJ) antibodies. The enhanced chemiluminescence (ECL; GE Healthcare) procedure was used to detect protein signals.

### Immunohistochemistry

Ki-67 expression was analyzed in paraffin-embedded sections obtained from IBC mouse tumors. Antigen retrieval was carried out using water bath in 0.01 M citrate buffer at pH 6.0. Slides were incubated for 2 h with 10% normal goat serum (Vector Labs, Burlingame, CA) with phenylhydrazine and Triton-X 100 and overnight with rabbit specific monoclonal antibody anti-Ki-67 (Epitomics, Burlingame, CA, dilution 1∶500). Sections were incubated with biotinylated goat anti-rabbit IgG (Sigma-Aldrich) diluted 1∶500 for 1 h, followed by the ABC reaction (Vector Laboratories, Burlingame, CA) overnight. For Vimentin expression, slides were incubated for 1 h with 5% normal goat serum (Vector Labs, Burlingame, CA) with 1X Tris Buffered-Saline and Tween 20 (TBST) and overnight with rabbit specific monoclonal antibody anti-Vimentin (D21H3) (Cell Signaling, Danvers, MA) diluted in SignalStain® Antibody Diluent (Cell Signaling, 1∶100).

### Statistical Analysis

Quantified data are expressed as mean ± S.E.M. Statistical analyses were done using GraphPad Prism version 5.0 b (San Diego, CA) or Microsoft excel. *In vitro* studies, the data were analyzed using regular analysis of variance procedures. Factors of interest always included treatment, time, and their interaction. For gene expression studies in SUM-149 vehicle, or 0.5 mg/mL Reishi treated cells were individually assessed using the 2^(−ΔCt)^ formula by comparing their relative gene expression to the expression of reference genes. The *P* values for gene expression PCR array analysis was calculated based on a Student’s t-test of the replicate 2^(−ΔCt)^ values for each gene in the control group and treatment groups following manufacturer’s instructions. Values *P*<0.05 were considered significant. *In vivo* tumor growth studies, *P* values were calculated using ANOVA and data was considered significant when *P*<0.05.

## Results

### Reishi Downregulates the Expression of Genes of the PI3K/AKT/mTOR Pathways

**Figure 1:**
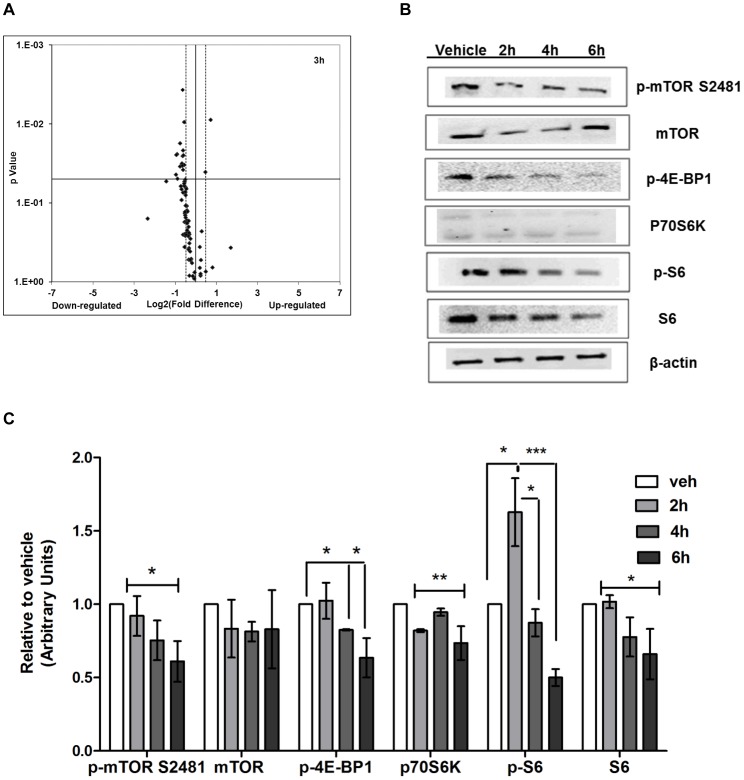
Reishi decreases the expression of PI3K/AKT signaling pathway genes and of mTORC1 effectors. A. Total SUM-149 cell RNA extraction was performed from three different experimental plates treated with 0 mg/mL (n = 3/vehicle) or 0.5 mg/mL Reishi (n = 3/treatment) for 3 hours. RT^2^ PCR arrays designed to profile the expression of PI3K/AKT pathway-specific genes were used according to manufacturer’s instructions (SA Biosciences). Volcano plot shows the effects on gene expression analyzed at −1.4≥1.4 log_2_-fold change (dashed lines). Down-regulated genes are to the left of the vertical black line while up-regulated genes are to the right. Statistically significant (*P*<0.05) regulated genes are above the horizontal black line. B. SUM-149 cells were grown in 5% FBS media for 24 hours prior to treatment with vehicle (0 mg/mL) or Reishi extract (0.5 mg/mL) for 2, 4, and 6 hours before lysis. Equal amount of protein from each sample was used for Western blot analysis with antibodies against mTORC1 effector proteins. C. Columns represent means ±SEM of integrated density units of protein, normalized to β-actin levels and shown relative to vehicle controls (without Reishi treatment). Statistically significant differences are shown at *P<0.05, **P<0.01, ***P<0.0001.

Using the established IBC cell model, SUM-149 cells, we previously published that Reishi selectively reduced cancer cell viability and invasion [9]. To test whether Reishi treatment affects the expression of genes specifically involved in the PI3K/AKT/mTOR pathway, we performed PI3K/AKT signaling RT^2^ Profiler PCR arrays in SUM-149 cells treated with vehicle or 0.5 mg/mL Reishi for 3 hours. As shown in **Figure 1A**, Reishi reduced the expression of most of the genes assayed in this signaling pathway. **Table 2** depicts genes in which the expression was significantly affected by Reishi treatment and that display a −1.4≥1.4 Log_2_ fold change. Of the genes that were statistically different (*P*<0.05), 19/21 were downregulated in expression by Reishi, including *AKT1, CCND1, EIF4GI, MAPK1, and HRAS*. The two genes that were significantly upregulated in expression were *JUN* and *FOS* by 1.7 and 1.4 fold, respectively. In addition, there were 10 additional genes that showed tendencies for downregulation by Reishi, depicted in **Table S1**. Because Reishi reduced the expression of *CCND1,* we also assessed the expression of additional cell cycle regulatory genes at pre-cell cycle (3 and 6 hours) and at post-cell cycle hours (24 and 48 hours) in SUM-149 cells treated with vehicle or 0.5 mg/mL. Although Reishi modulated the expression of these genes at various time points, Reishi significantly reduced the expression of *CCNA2* and *CCNB2* after 48 hours of treatment by −3.5 and −5.0 fold, respectively (**figure S1**). The modulatory effects of Reishi on cell cycle progression in IBC cells are consistent with its downregulation of mTOR signaling and the activation (reduced phosphorylation) of 4E-BP1.

**Table 2:** *In vitro* effects of 0.5 mg/mL Reishi on the expression of PI3K/AKT/mTOR pathway genes.

Gene	Complete name	Fold change	*P* value
*ADAR*	Adenosine deaminase, RNA-specific	−1.5	0.04
*AKT1*	V-akt murine thymoma viral oncogene homolog 1	−1.7	0.03
*BAD*	BCL2-associated agonist of cell death	−1.5	0.02
*CDC42*	Cell division cycle 42 (GTP binding protein, 25 kDa)	−1.5	0.05
*CCND1*	Cyclin D1	−2.7	0.05
*EIF4G1*	Eukaryotic translation initiation factor 4 gamma, 1	−1.9	0.02
*FOS*	FBJ murine osteosarcoma viral oncogene homolog	1.4	0.04
*GRB2*	Growth factor receptor-bound protein 2	−1.9	0.05
*HRAS*	V-Ha-ras Harvey rat sarcoma viral oncogene homolog	−1.9	0.02
*HSPB1*	Heat shock 27 kDa protein 1	−1.5	0.03
*ITGB1*	integrin, beta 1	−1.4	0.05
*JUN*	Jun oncogene	1.7	0.009
*MAPK1*	Mitogen-activated protein kinase 1	−1.6	0.03
*MAPK14*	Mitogen-activated protein kinase 14	−1.5	0.05
*MYD88*	Myeloid differentiation primary response gene (88)	−1.5	0.03
*PIK3R1*	Phosphoinositide-3-kinase, regulatory subunit 1 (alpha)	−2.0	0.04
*PTPN11*	Protein tyrosine phosphatase, non-receptor type 11	−1.6	0.03
*SHC1*	Src homology 2 domain containing transforming protein 1	−1.5	0.0095
*SRF*	Serum response factor	−1.6	0.03
*TOLLIP*	Toll interacting protein	−1.7	0.02
*YWHAH*	Tyrosine 3-monooxygenase/tryptophan 5-monooxygenase activation protein, eta polypeptide	−1.6	0.004

Only genes that demonstrated −1.4>1.4 log_2_-fold difference and P<0.05 from RT^2^ PCR arrays are shown.

### Reishi Regulates the Expression of mTOR Effector Proteins

We previously showed that Reishi reduces the expression of IBC biomarkers, including E-cadherin and eIF4G at the protein level after 24 h of treatment [9]. In order to determine whether Reishi compromises the expression of mTOR and its downstream effectors at early time points, we conducted western blot analysis using cell lysates from cells treated for 2, 4 or 6 hours with 0.5 mg/mL Reishi. As shown in **Figure 1B**, Reishi significantly reduced the expression of pmTOR at Ser2481 (*P*<0.05). mTOR Ser(P)-2481 promotes mTOR intrinsic catalytic activity in both mTORC1 and mTORC2 complexes [23]. Reishi downregulation of mTOR was also manifested on downstream effector signaling, as p70S6K, S6, p-S6 and p-4E-BP1 levels were all reduced by Reishi compared to vehicle treated cell lysates (**Figure 1B, 1C**). We also investigated whether Reishi affects the expression of Akt and its phosphorylation at serine 473. As shown in **figure S2**, Akt activity or total protein expression levels were not affected by Reishi treatment.

### Reishi Reduces eIF4F Complex Levels and Protein Synthesis in IBC Cells

**Figure 2:**
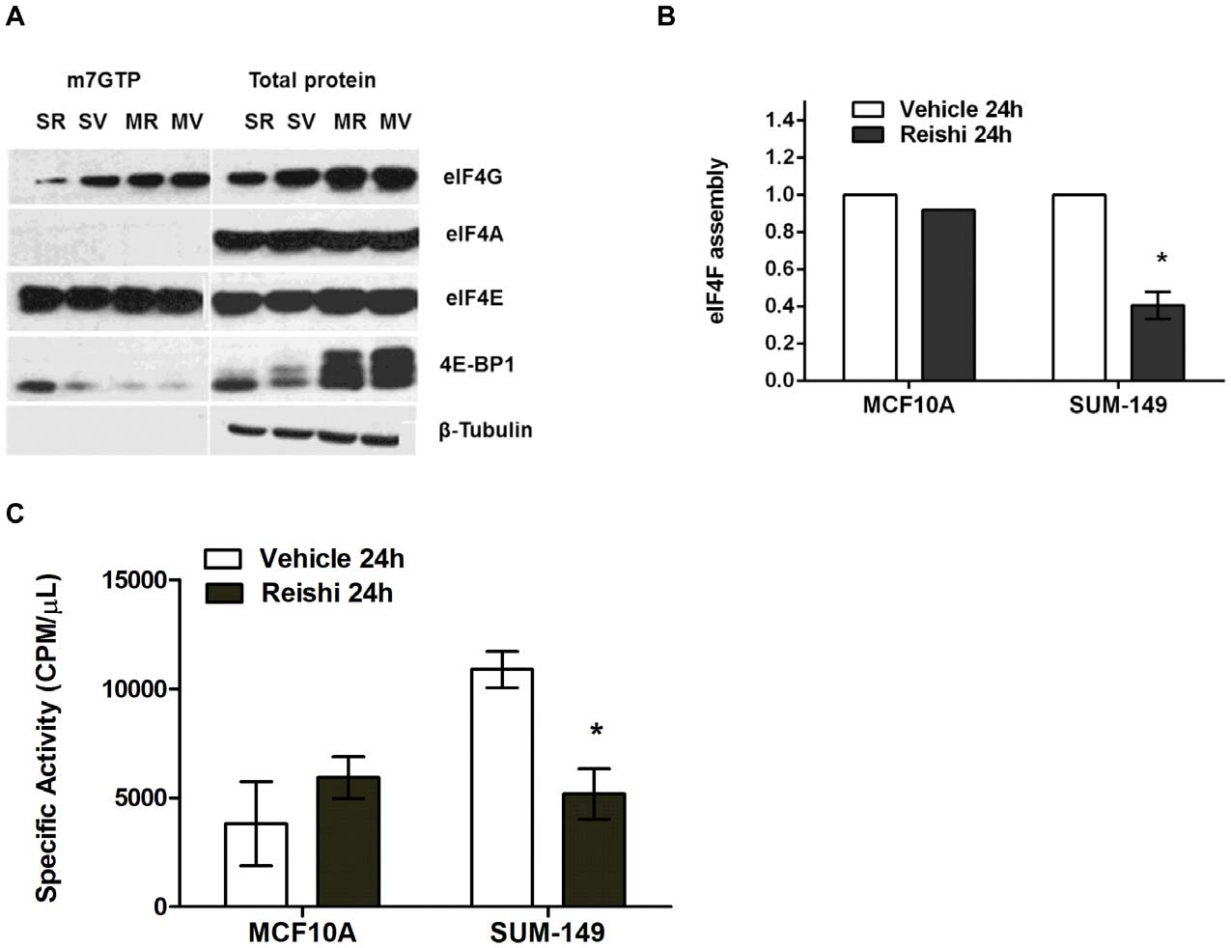
Reishi decreases EIF4F complex levels and protein synthesis in IBC cells. A. SUM-149 and MCF10A cells were treated with vehicle (0mg/mL, SV or MV) or 0.5mg/mL Reishi (SR or MR) for 24 hours before lysis. Western blot analyses were completed for total eIF4G, eIF4A, eIF4E, and 4E-BP1 obtained from m7GTP pull-down lysates and whole cell lysates. B. Graph represents quantification of eIF4F complex as in Dumstorf et al., 2010 [25], where eIF4G normalized to eIF4E is divided by 4E-BP1 normalized to eIF4E [(eIF4G/eIF4E)/(4E-BP1/eIF4E)]. Number of biological replicates (n) varies among experiments (SUM-149; n = 3, MCF10A; n = 1). Columns show means ± SEM of integrated density units, shown relative to vehicle controls. Reishi significantly reduces eIF4F complex assembly at *P<0.02 in IBC SUM-149 cells. C. 1×10^5^ cells (SUM-149 and MCF10A) were seeded per well in a six well plate and treated with vehicle or 0.5 mg/mL Reishi for 24 hours. The treatment was removed and the cells were re-incubated for 30 minutes in methionine/cysteine - free DMEM. L-[^35^S] Methionine and L-[^35^S] Cysteine (2 mCi/mL) +2% FBS was then added to the cultures. Total cell lysates prepared in NP-40 lysis buffer were analyzed for incorporated radioactivity in trichloroacetic acid precipitates. Data are expressed as means ± SEM of duplicate determinations. Experiment was repeated three times. Reishi significantly (*P<0.05) reduces protein synthesis by 48%.

Reishi reduced 4E-BP1 phosphorylation at early timepoints (**Figure 1B**) and eIF4G levels by 24 hours post-treatment [9]. As the hypophosphorylation of 4E-BP1 increases the binding of 4E-BP1 to eIF4E [24], thereby diminishing eIF4F complex levels, we sought to determine whether Reishi would increase this association using m7GTP cap analog beads to capture eIF4E and to pull down associated proteins as described in [25]. Accordingly, we found that at 24 hours post-Reishi treatment the amount of 4E-BP1 bound to eIF4E increases in SUM-149 cells (**Figure 2A**). To quantify this, we normalized the levels of co-captured 4E-BP1 and eIF4G to eIF4E and then divided the normalized eIF4G values by the normalized 4E-BP1 values after m7GTP co-capture in MCF10A and SUM-149 cells (**Figure 2B**). eIF4F translation initiation complex assembly levels in SUM-149 cells, are significantly (∼60%) reduced in Reishi treated cells (*P*<0.02). Interestingly, this effect was not observed in Reishi treated non-cancerous mammary epithelial MCF10A cells, or in IBC cells treated with or without Reishi for 4 (data not shown) or 6 hours (**figure S3**). Thus, disruption of eIF4F translation initiation complex levels, unlike downregulation of mTOR, required an extended period of treatment.

Next, we investigated the ability of Reishi to inhibit global protein synthesis by performing metabolic labeling of protein synthesis activity using ^35^S-methionine/cysteine in SUM-149 IBC cells. As shown in **Figure 2C**, Reishi significantly (*P*<0.05) reduced protein synthesis by half in IBC cells, an effect not seen in Reishi treated MCF10A cells. Therefore, these results support the hypothesis that Reishi has a selective anti-cancer effect that is manifested by downregulating the mTOR pro-survival pathway and ultimately protein synthesis.

### Reishi Exhibits Anti-tumor Effects in vivo

**Figure 3:**
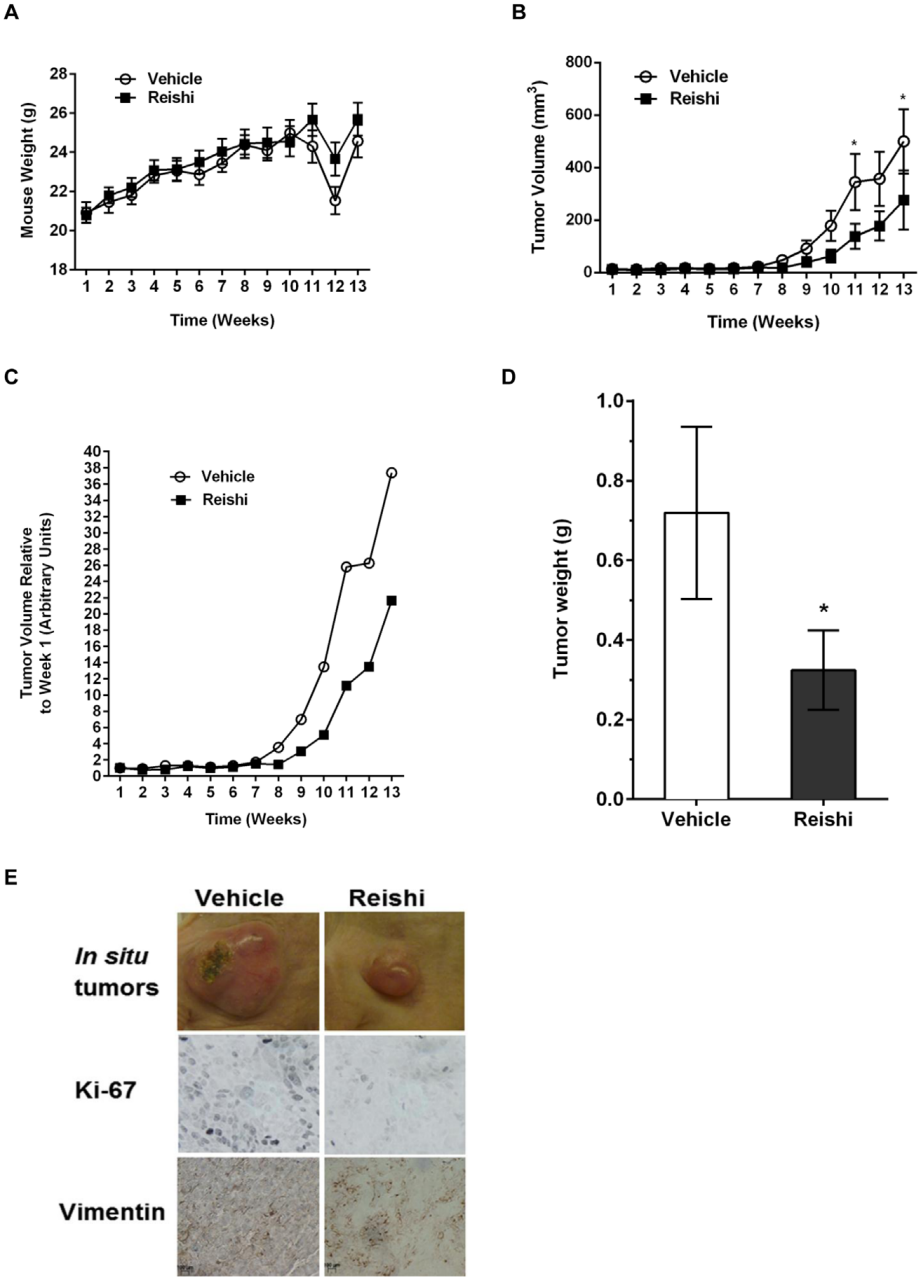
Reishi reduces tumor growth, tumor weight, and proliferative and mesenchymal marker expression. 1.5×10^6^ cells/100µL of SUM-149 cells, were injected into the mammary fat pad of severe combined immunodeficient (SCID) mice. One week following injection, mice were orally gavaged with vehicle, (n = 11) or 28 mg/kg BW Reishi (n = 11) daily for a period of 13 weeks. A. Mice weights were recorded weekly. There were no differences in body weights of mice that received Reishi compared to animals that received vehicle control. B. Tumor volume was recorded weekly using caliper measurements, and measured as described in materials and methods. C. Average tumor volume measurements per week from mice treated with vehicle or Reishi were normalized relative to the average tumor volume measurements from mice treated with vehicle or Reishi obtained at week one. Reishi significantly reduces tumor growth by 58% (*P*<0.02). D. Tumor weights were obtained at the end of the study. Columns show means ± SEM. Reishi significantly (*P<0.05) reduces tumor weight by 45%. E. Tumors were excised on the 13^th^ week post Reishi, fixed in 10% formalin and embedded in paraffin before immunostaining with antibodies against Ki-67 and vimentin. Reishi treated tumors show reduced size, lower Ki-67 and Vimentin protein expression.

Because Reishi selectively reduces cancer cell viability and invasion, as well as the expression of key proteins involved in the pathogenesis of IBC cells [9] we sought to determine the in vivo efficacy of this extract in SCID mice injected with SUM-149 IBC cells. Mice were injected with IBC cells in Matrigel in their 4th mammary fat pad. When tumors were palpable (∼ one week post-injection), mice were orally gavaged daily with 0 or 28 mg/kg BW Reishi. This concentration is twice the recommended Reishi dose/body weight (1000 mg/daily) for an average adult woman (70 kg). Throughout 13 weeks, the mice were weighed weekly and tumor growth was recorded by precision caliper measurements. There were no differences in body weights (**Figure 3A**) or food consumption (data not shown) in mice that received Reishi compared to animals that received the vehicle control, which demonstrates that Reishi treatment is not toxic to mice. The drop in body weight at 12 weeks detected in both groups was a result of changing the weighing instrument for that week. However, the mice show similar body weights regardless of this change. In contrast, tumor volume was significantly (>50%) reduced (*P*<0.02) in the Reishi treated mice compared with mice gavaged daily with vehicle treatment (**Figure 3B, 3C**). At the end of 13 weeks of daily Reishi treatment, the mice were euthanized and primary tumors and spleens were weighed and collected for subsequent analysis. Reishi treated mice showed a 45% (*P*<0.05) lower tumor weight values (**Figure 3D**), while no changes were detected in spleen weights (data not shown). Part of the primary tumor was stored in 10% formalin for tissue paraffin block preparation and subsequent immunohistochemistry, another part was stored in RNAlater™ for real time RT^2^ profiler PCR array analysis and another part was flash frozen for subsequent western blot analysis of tumor tissue lysates. As depicted in **Figure 3E** **,** Reishi treated tumors showed reduced size accompanied by reduced levels of Ki-67 and Vimentin (cell proliferation and mesenchymal markers, respectively) when compared with tumors from mice receiving vehicle treatment. These data are consistent with the results of **Figure 1** demonstrating reduced mTOR activity in Reishi treated cancer cells.

**Figure 4:**
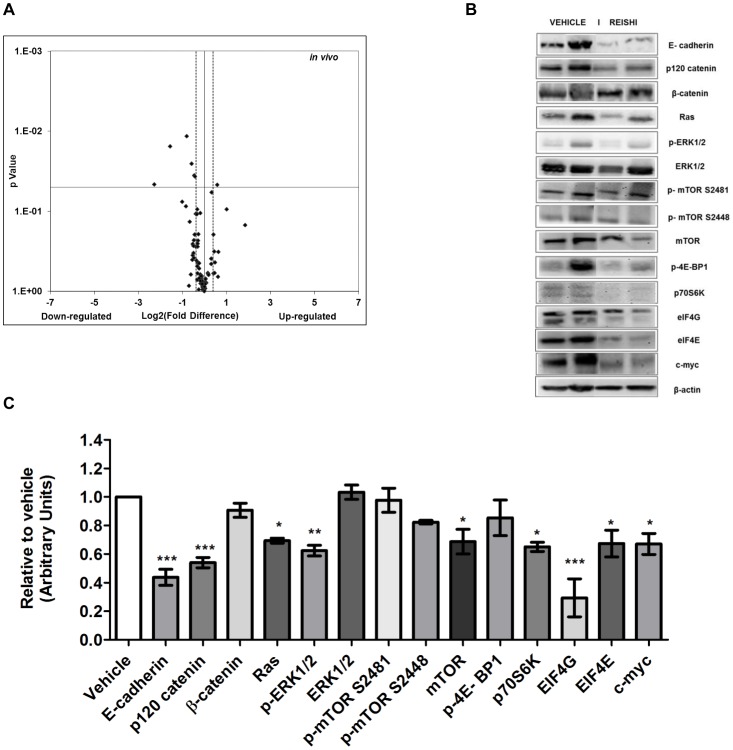
Reishi reduces PI3K/AKT/mTOR and MAPK pathway gene and protein expression. A. RT^2^ PCR array designed to profile the expression of PI3K/AKT pathway-specific genes was performed using 500 ng of tumor extracted RNA, according to manufacturer’s instructions (SA Biosciences). Volcano plot show effects on gene expression analyzed at −1.3≥1.3 log_2_-fold change (dashed line). Down-regulated genes are to the left of the vertical black line while up-regulated genes are to the right. Statistically significant regulated genes are above the horizontal black line at *P*<0.05. B. Equal amount of protein from each sample was used for western blot analysis with antibodies against key IBC proteins. Each lane depicts a representative tumor lysate from a different mouse of either vehicle or Reishi treatment. C. Quantification was done using integrated density units, normalized to β-actin and relative to vehicle. Columns show means ± SEM. Reishi downregulates the expression of key IBC proteins in vivo. *P<0.05, **P<0.01.

Total RNA was extracted from tumor lysates and PI3K/AKT/mTOR PCR arrays were conducted to determine Reishi effects on genes involved in this pro-survival pathway. As shown in **Figure 4A**, Reishi reduced the expression of 64% of the genes in the PCR array. Reishi significantly reduced the expression of 5 genes, including the eukaryotic initiation factor 4B and ribosomal protein S6 kinase, 70 kDa, polypeptide 1 (*EIF4B, RPS6KB1),* gap junction protein alpha 1, 43 kDa (*GJA1),* the pro-invasion gene encoding the p21 protein (cdc42/Rac)-activated kinase 1 *(PAK1),* and pyruvate dehydrogenase kinase, isozyme 1 *(PDK1)* (**Table 3**), while it increased the expression of the nuclear factor of kappa light polypeptide gene enhancer in B-cells inhibitor, alpha (*NFKBIA*). Additional genes affected by Reishi that show strong statistical tendencies are listed in **Table S2**. Moreover, to assess Reishi anti-IBC effects in vivo, we examined the expression of various proteins in tumor lysates. First we assessed the effects of Reishi on key IBC proteins. As shown in **Figures 4B & 4C**, Reishi reduced the expression of IBC biomarker, E-cadherin, and two proteins in which their mRNAs are translated in an IRES-dependent manner, p120-catenin, and c-myc. Next, we examined the in vivo effects of Reishi on mTOR signaling proteins, where Reishi significantly reduced the expression of mTOR, p70S6K, and eIF4G. However, the total expression or activation of Akt was not affected by the treatment (**figure S4**). Because loss of mTOR function has an impact on MAPK activation status [26], we verified if Reishi activates the MAPK pathway. Herein we show that Reishi reduces the expression of RAS, and of p-ERK1/2 without affecting total ERK1/2 levels (**Figure 4B, 4C**). These results provide evidence that the various compounds found in Reishi, which have yet to be isolated, have an inhibitory anti-cancer effect manifested by reduced tumor growth, gene expression, protein synthesis and concomitant inhibition of the mTOR and MAPK pathways showing relevant therapeutic implications in IBC. This study provides compelling reason to pursue further purification and isolation of these compounds.

**Table 3:** *In vivo* effects of Reishi on the expression of PI3K/AKT/mTOR pathway genes.

Gene	Complete name	Fold change	*P* value
*EIF4B*	Eukaryotic translation initiation factor 4B	−1.4	0.03
*GJA1*	Gap junction protein, alpha 1, 43kDa	−2.9	0.01
*NFKBIA*	Nuclear factor of kappa light polypeptide gene enhancer in B-cells inhibitor, alpha	1.5	0.04
*PAK1*	p21 protein (Cdc42/Rac)-activated kinase 1	−1.3	0.03
*PDK1*	Pyruvate dehydrogenase kinase, isozyme 1	−1.7	0.01
*RPS6K1*	Ribosomal protein S6 kinase,70kDa,polypeptide 1	−1.9	0.02

Only genes that demonstrated −1.3>1.3-log_2_ fold difference and P<0.05 from RT^2^ PCR arrays are shown.

## Discussion

The PI3K/AKT/mTOR network plays a key regulatory function in cell survival, proliferation, migration, metabolism, angiogenesis, and apoptosis [27]. Genetic aberrations such as loss of *PTEN* as in SUM-149 cells used herein make this pathway one of the most commonly disrupted in human breast cancer. The common activation of the PI3K pathway in breast cancer has led to the development of compounds targeting the downstream effector, mTOR. The influences of other oncogenic pathways such as MAPK on the PI3K pathway and the known feedback mechanisms of activation have prompted the testing and development of compounds with broader effect at multiple levels to obtain a more potent antitumor activity and possibly a meaningful clinical effect. Our results show that Reishi exhibits these properties, as it affects the expression of various proteins of the PI3K/AKT/mTOR pathway.

We previously reported that treatment of inflammatory breast cancer cells with a commercially available extract consisting of 13% polysaccharides, 6% triterpenes and 1% cracked spores of *Ganoderma lucidum* (Reishi) for 24 hours resulted in viability and invasion inhibition, tumor spheroid disruption, apoptosis induction and downregulation of key genes and proteins important in IBC [9]. Herein, we investigated Reishi’s effects at early timepoints using the triple negative, *PTEN* null, IBC cell line SUM-149 as an *in vitro* model, and for longer treatment times using an *in vivo* mouse model, focusing on mTORC1 downstream signaling effectors.

We show that Reishi modulatory effects begin to occur as early as 3 hours post-treatment at the level of mRNA expression/abundance, where 74/84 (88%) of the genes were downregulated and 19/84 (23%) were significantly downregulated in SUM-149 cells. Moreover, Reishi was found to compromise the protein expression of mTORC1 effectors. We show that the activity of mTOR through surrogate Ser(P)-2481 phosphorylation is reduced in Reishi treated cells, an effect not seen in total mTOR levels. Studies showed that mTOR Ser(P)-2481 is activated in a wortmannin-sensitive manner in both mTORC1 and mTORC2 complexes, demonstrating a requirement for PI3K in mTORC1 and mTOR2 autophosphorylation. Moreover, the level of mTORC1-associated mTOR Ser(P)-2481 correlates positively with the extent of mTORC1 signaling [23]. Herein we show that Reishi reduces *PI3KR1 and PI3KR2* gene expression, which code for PI3K regulatory subunits α and β. In addition, we show that Reishi extract also affects the expression of the best-characterized mTORC1 substrates, p70S6K and 4E-BP1. One of the consequences of Reishi’s modulatory effect is protein synthesis inhibition, which we also demonstrate to act by inhibiting cap-dependent translation (reduced eIF4F complex levels), and globally by reducing by half the levels of protein synthesis in IBC cells but not in non-cancerous mammary epithelial cells. Studies silencing eIF4G in SUM-149 IBC cells show reduced eIF4G protein levels by at least 90%, compared with control cells, but with only a slight reduction in overall protein synthesis (15%), no effect on cell viability, and only slightly impaired cell proliferation [17]. Therefore, our results suggest that Reishi’s anti-cancer effect is not exclusively through eIF4G downregulation, but likely occurs through an inhibitory combination of signaling pathways that include PI3K and mTOR pathways and on levels/activity of eIF4F complex proteins.

Reishi significantly upregulated *JUN* and *FOS* by 1.7 and 1.4 fold, respectively. However, these transcription factors are regulated at various levels including transcriptionally, via mRNA stability, post-translational modifications such as phosphorylation and by protein turnover. Active Fos proteins dimerize with Jun proteins to form Activator Protein-1 (AP-1), a transcription factor that binds to TRE/AP-1 elements and activates transcription of genes such as cyclin D1, which herein we show is modulated by Reishi treatment. Moreover, studies show that the same Reishi extract as the one used in the current study inhibits AP-1 and NF- κB transcriptional activation in MDA-MB-231 breast cancer cells [7], [28]. Contrary to Jiang et al. results, where they show that Reishi inhibits Akt expression in MDA-MB-231 breast cancer cells [28] our study shows that in IBC cells, the same Reishi extract reduces *AKT1* gene levels, but not Akt protein expression or phosphorylation. Independently, Reishi inhibitory effects on mTOR signaling and IBC progression are evident.

Our *in vivo* studies show that Reishi treated mice have statistically significantly reduced tumor growth and tumor weight. Previous studies have shown that individual components from Reishi, such as specific polysaccharides or triterpenes inhibit invasion and metastasis in various xenograft models [29], [30]. However, our study is the first to show that an extract containing a combination of polysaccharides and triterpenes derived from the Reishi mushroom has anti-tumor growth effects in a very aggressive type of cancer. Even though the concentration of Reishi extract that was required to demonstrate a significant difference in tumor growth is higher than the current concentration suggested for humans, the concentration used was not toxic to the mice. Herein, we show that Reishi-treated mice displayed a disparate effect on tumor growth starting at week eight of treatment, and by the end of the 13^th^ week of the study the difference in tumor growth was almost 60%. The lag time in tumor growth observed for the first eight weeks was seen in both vehicle and Reishi treated animals. This is due to the unique phenotype of IBC, where SUM-149 cells tend to form a “diffuse” tumor during the first weeks post-inoculation. This phenomenon was reported in a similar study where caliper measurements could not be initiated until 30 days following SUM-149 cell inoculation at the mammary fat pad of SCID mice [31]. This tumorigenic effect also resembles IBC tumor formation in women, where most present without a palpable mass at the time of diagnosis and then proliferates at high rates [32]. At the end of the study, mouse tumors were excised and weighed; showing that Reishi treated tumors weighed 45% less than vehicle controls. Necrotic centers were observed equally in some vehicle and Reshi treated mice (data not shown). Reishi treated SUM-149 tumors showed a decrease in the levels of the Ki-67 cell proliferation and Vimentin mesenchymal markers compared with those from the control treatment. We previously reported that although E-cadherin expression is compromised by Reishi treatment in SUM-149 cells, β-catenin does not translocate into the nucleus [9]. In this study, we confirm that although E-cadherin expression is reduced with Reishi treatment in vivo, epithelial to mesenchymal transition does not occur as shown by lower Vimentin levels in the tumors, and neither do we see a subsequent increase in proliferation due to a potential β-catenin translocation into the nucleus, as demonstrated by lower proliferation rates shown by reduced Ki-67 tumor levels.

The anti-tumor effects of Reishi at the molecular level depicted here may be the result of different compounds within the Reishi extract that are affecting and targeting various signaling pathways simultaneously. We show that Reishi treated tumors reduce the expression at both the gene and protein level of important molecules in the PI3K/AKT/mTOR and MAPK signaling pathways. Specifically, Reishi affects mTOR levels and therefore, activity. This result is in contrast to that seen *in vitro*. However, our *in vitro* studies presented here show Reishi treatment at early timepoints, thus it will be interesting to study the effects of Reishi on mTOR activation at the cellular level at longer exposure times to determine if it mimics the in vivo effects. In IBC SUM-149 cells, the PI3K/AKT/mTOR signaling pathway is elevated, due to the lack of *PTEN*. Herein we demonstrated that Reishi downregulates the expression of molecules involved in this pathway, effectively circumventing the *PTEN* null effect. Moreover, our study shows that Reishi reduced the activation of the parallel Ras/Raf kinase/extracellular signal-regulated (ERK) pathway via p-ERK 1/2 (Thr 202/Tyr 204) and reduced Ras levels. ERK1/2 synergizes with the mTOR pathway in the activation of p70S6K, the downstream effector of p-p70S6K in the form of p-S6 expression [33], [34], and on the phosphorylation inactivation of 4E-BP1 [35], [36], which we also find to be downregulated by Reishi. Moreover, there seems to be an impact of convergent signaling on cell cycle progression as evidenced by a decrease in the Ki-67 proliferation marker.

In this study we also show that in vivo E-cadherin and p120-catenin downregulation occur together with reduced levels of eIF4G, confirming and extending our previous findings [9]. Here we show that downregulation of these proteins contribute to reduced tumor growth. Similar results were found by Silvera and collaborators where eIF4G silencing results in less p120-catenin mRNA translation and subsequent E-cadherin cytoplasmic re-localization, disrupting tumor spheroid formation that is necessary for IBC invasion [17]. Based on our findings, we conclude that Reishi is an anti-cancer agent that selectively affects gene and protein expression and therefore, activity of molecules involved on cancer cells and shows tumor inhibitory effects. This action can be correlated with reduced levels of key signaling pathways that ultimately increase cancer cell growth, proliferation and survival. To date, effects of Reishi extract have not been tested on IBC in vivo models or patients. Studies are being conducted in vivo to test the efficacy of Reishi in IBC using this SCID mouse model in combination with conventional therapy and *in vitro* at longer exposure times. Therefore, our findings suggest that Reishi extract could be used as a novel anticancer therapeutic for IBC patients.

## Supporting Information

Figure S1 **Effect of Reishi in the expression of cell cycle regulatory genes.** Total SUM-149 cell RNA extraction was performed from three different experimental plates treated with 0 mg/mL (n = 3/vehicle) or 0.5 mg/mL Reishi (n = 3/treatment) for 3, 6, 24 or 48 hours. Down-regulated genes are below the horizontal black line while up-regulated genes are above. Columns show means. Statistically significant differences are shown at *P<0.05.
(TIF)

Figure S2 **Effect of Reishi in the expression of Akt** ***in vitro*** **.** A. SUM-149 cells were grown in 5% FBS media for 24 hours prior to treatment with vehicle (0 mg/mL) or Reishi extract (0.5 mg/mL) for 2, 4, and 6 hours before lysis. Equal protein concentration from each sample was used for Western blot analysis with antibodies against total and phosphorylated Akt. B. Columns represent means ± SEM of integrated density units of protein, normalized to β-actin levels and shown relative to vehicle controls (without Reishi treatment).
(TIF)

Figure S3 **EIF4F complex levels after 6** **h of Reishi treatment in IBC SUM-149 cells.** SUM-149 cells were incubated with vehicle (0 mg/mL) or 0.5 mg/mL Reishi for 6 h before lysis. Graph represents eIF4G normalized to eIF4E divided by 4E-BP1 normalized to eIF4E [(eIF4G/eIF4E)/(4E-BP1/eIF4E)] as in [25]. Columns show means ± SEM. Reishi does not affect eIF4F complex assembly at 6 h of treatment.
(TIF)

Figure S4 **Effect of Reishi in the expression of Akt** ***in vivo*** **.** Equal amount of protein from each sample was used for western blot analysis with antibodies against total and phosphorylated Akt.
(TIF)

Table S1 *In vitro* expression patterns of PI3K/Akt pathway genes. This table includes all genes that show tendency to be significantly up- or down- regulated with 0.5 mg/ml Reishi at a *P* value between 0.06 and 0.08 when where compared to vehicle controls. See Table 1 for genes that are significantly regulated and are analyzed at −1.4≥1.4 log_2_-fold changes.
(DOCX)

Table S2 *In vivo* expression patterns of PI3K/Akt pathway genes. This table includes all genes that show tendency to be significantly up- or down- regulated with 0.5 mg/ml Reishi at a *P* value between 0.06 and 0.08 when where compared to vehicle controls. See Table 2 for genes that are significantly regulated and are analyzed at −1.3≥1.3 log_2_-fold changes.
(DOCX)
