# Supplementary figures and images for: Anti-Tumor Effects of Ganoderma lucidum (Reishi) in Inflammatory Breast Cancer in In Vivo and In Vitro Models
Source: PLoS One. 2013 Feb 28;8(2):e57431. doi: 10.1371/journal.pone.0057431 (PMC3585368; doi:10.1371/journal.pone.0057431)

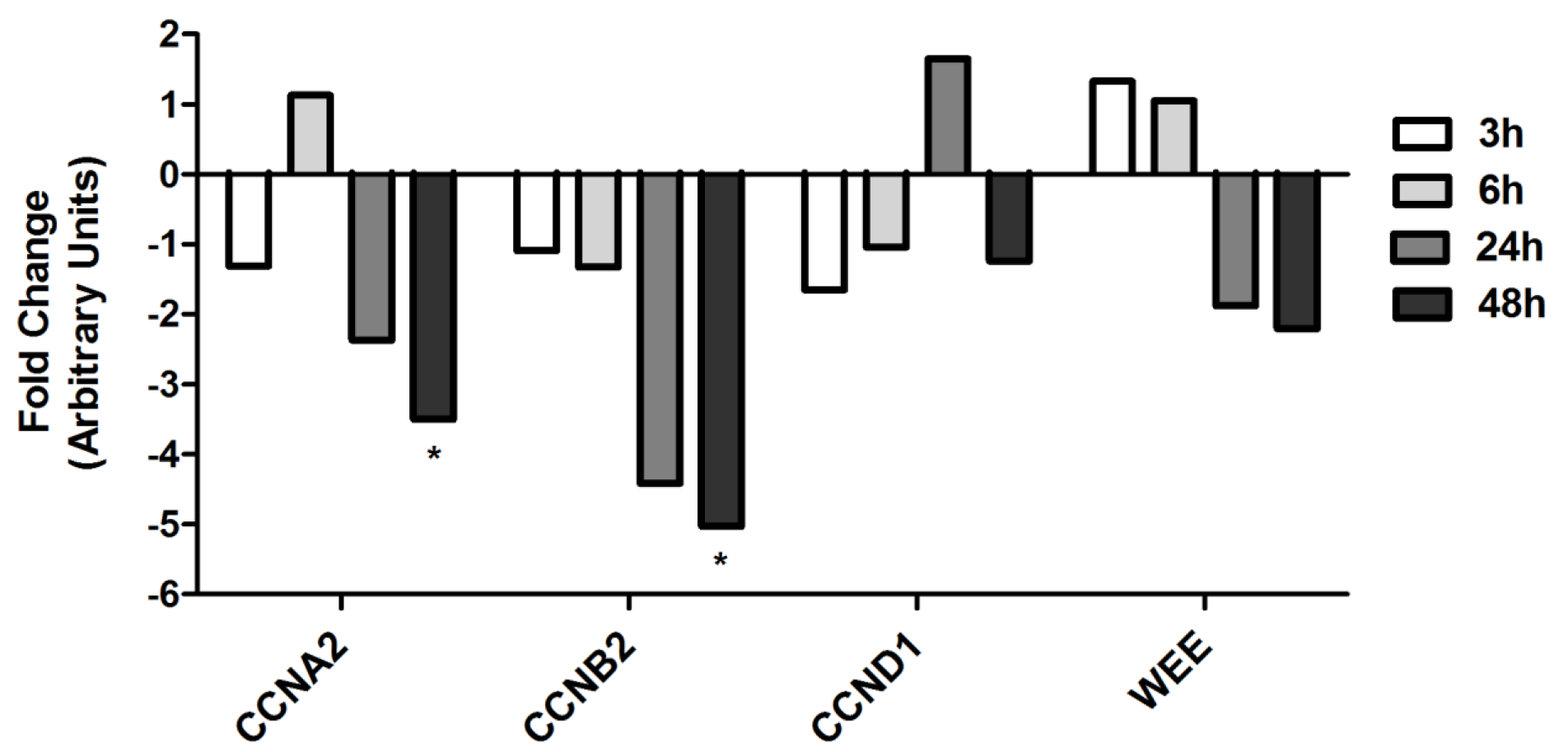

Supplement: Figure S1 — Effect of Reishi in the expression of cell cycle regulatory genes. Total SUM-149 cell RNA extraction was performed from three different experimental plates treated with 0 mg/mL (n = 3/vehicle) or 0.5 mg/mL Reishi (n = 3/treatment) for 3, 6, 24 or 48 hours. Down-regulated genes are below the horizontal black line while up-regulated genes are above. Columns show means. Statistically significant differences are shown at *P<0.05. (TIF) [file pone.0057431.s001.tif]

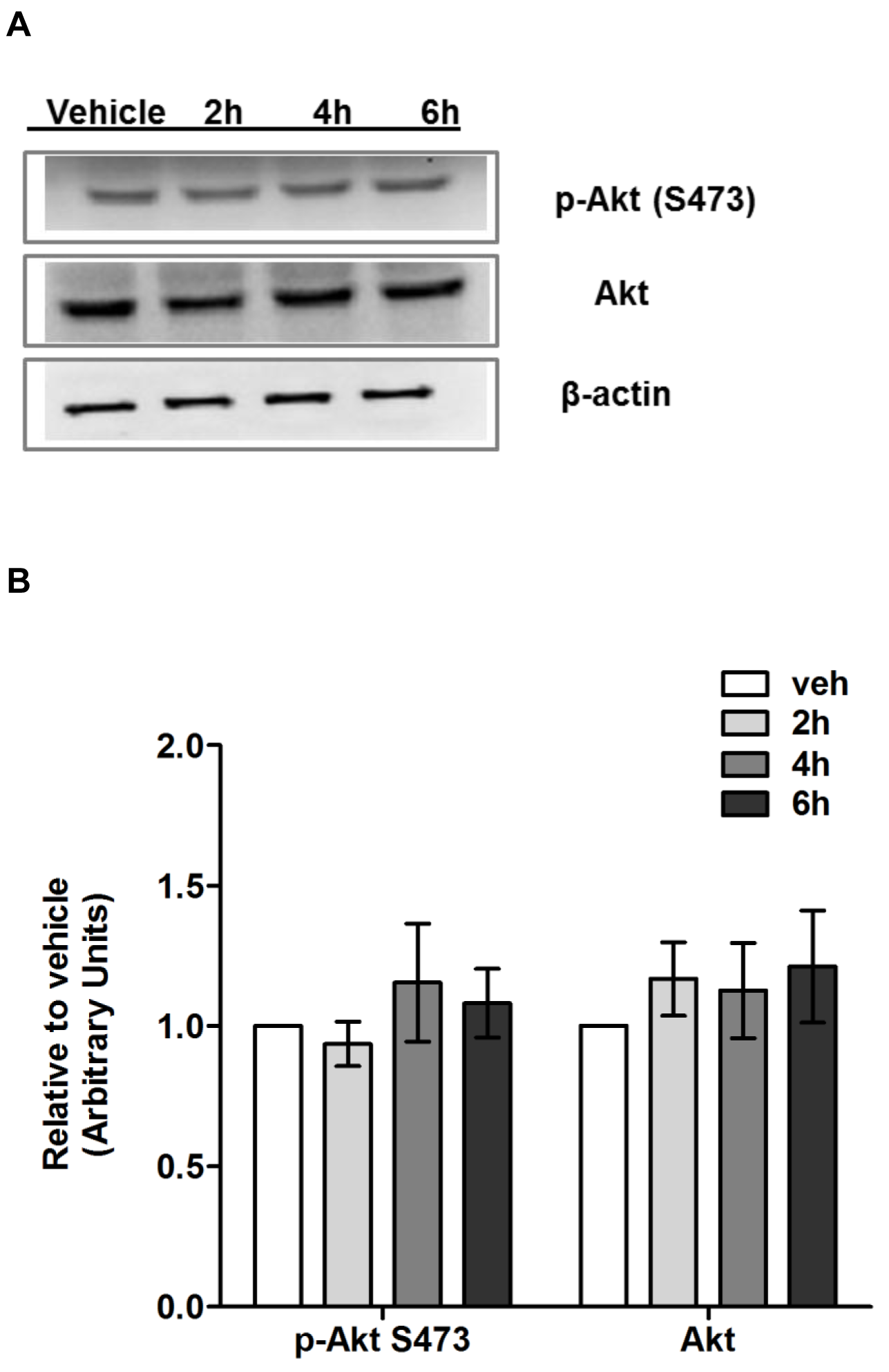

Supplement: Figure S2 — Effect of Reishi in the expression of Akt in vitro . A. SUM-149 cells were grown in 5% FBS media for 24 hours prior to treatment with vehicle (0 mg/mL) or Reishi extract (0.5 mg/mL) for 2, 4, and 6 hours before lysis. Equal protein concentration from each sample was used for Western blot analysis with antibodies against total and phosphorylated Akt. B. Columns represent means ± SEM of integrated density units of protein, normalized to β-actin levels and shown relative to vehicle controls (without Reishi treatment). (TIF) [file pone.0057431.s002.tif]

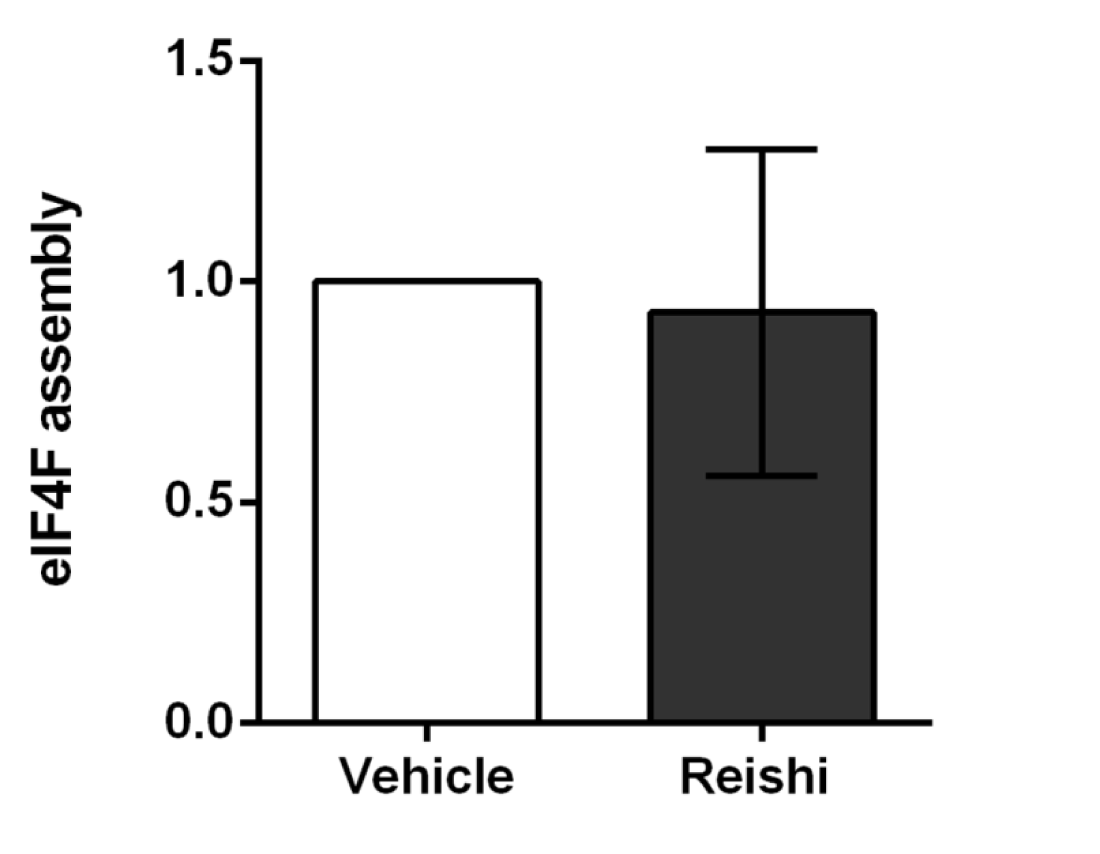

Supplement: Figure S3 — EIF4F complex levels after 6 h of Reishi treatment in IBC SUM-149 cells. SUM-149 cells were incubated with vehicle (0 mg/mL) or 0.5 mg/mL Reishi for 6 h before lysis. Graph represents eIF4G normalized to eIF4E divided by 4E-BP1 normalized to eIF4E [(eIF4G/eIF4E)/(4E-BP1/eIF4E)] as in [25]. Columns show means ± SEM. Reishi does not affect eIF4F complex assembly at 6 h of treatment. (TIF) [file pone.0057431.s003.tif]

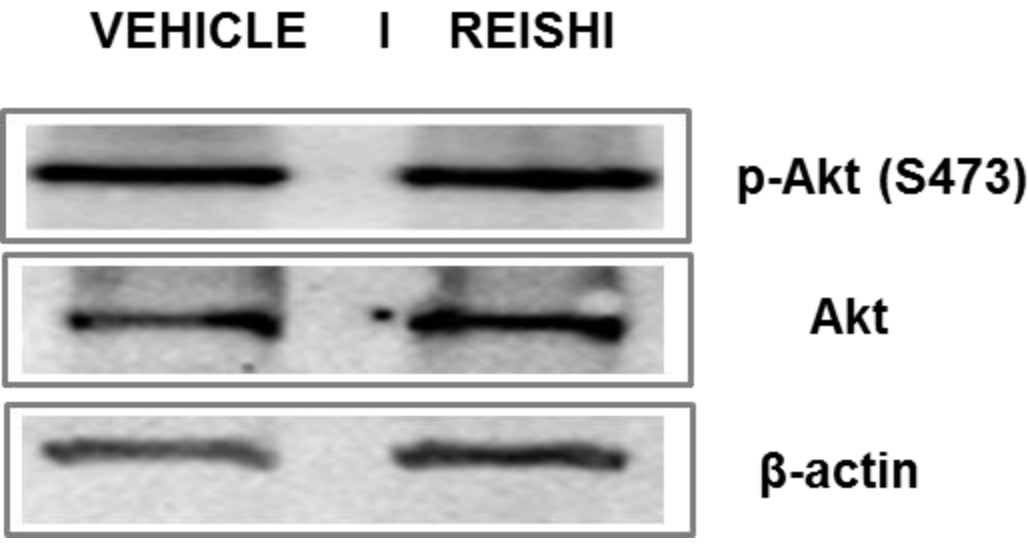

Supplement: Figure S4 — Effect of Reishi in the expression of Akt in vivo . Equal amount of protein from each sample was used for western blot analysis with antibodies against total and phosphorylated Akt. (TIF) [file pone.0057431.s004.tif]
