# Supplementary material for: Anti-Tumor Effects of Ganoderma lucidum (Reishi) in Inflammatory Breast Cancer in In Vivo and In Vitro Models
Source: PLoS One. 2013 Feb 28;8(2):e57431. doi: 10.1371/journal.pone.0057431 (PMC3585368; doi:10.1371/journal.pone.0057431)
Supplement: Table S1 — In vitro expression patterns of PI3K/Akt pathway genes. This table includes all genes that show tendency to be significantly up- or down- regulated with 0.5 mg/ml Reishi at a P value between 0.06 and 0.08 when where compared to vehicle controls. See Table 1 for genes that are significantly regulated and are analyzed at −1.4≥1.4 log2-fold changes. (DOCX) [file pone.0057431.s005.docx]

**Table S1. *In vitro* expression patterns of PI3K/Akt pathway genes.**

| **Gene symbol** | **Complete name** | **Fold change** |
| --- | --- | --- |
| *AKT3* | V-akt murine thymoma viral oncogene homolog 3 (protein kinase B, gamma) | -1.4 |
| *CTNNB1* | Catenin (cadherin-associated protein), beta 1, 88kDa | -1.5 |
| *EIF4E* | Eukaryotic translation initiation factor 4E | -1.4 |
| *IGF1R* | Insulin-like growth factor 1 receptor | -1.5 |
| *ILK* | Integrin-linked kinase | -1.4 |
| *PDPK1* | 3-phosphoinositide dependent protein kinase-1 | -1.6 |
| *PIK3R2* | Phosphoinositide-3-kinase, regulatory subunit 2 (beta) | -1.4 |
| *RAF1* | V-raf-1 murine leukemia viral oncogene homolog 1 | -1.4 |
| *TSC1* | Tuberous sclerosis 1 | -1.5 |
| *TSC2* | Tuberous sclerosis 2 | -1.7 |

This table includes all genes that show tendency to be significantly up- or down- regulated with 0.5mg/ml Reishi at a P value between 0.06 and 0.08 when where compared to vehicle controls. See Table 1 for genes that are significantly regulated and are analyzed at -1.4 ≥ 1.4 log_2_-fold changes.
