# Supplementary material for: Anti-Tumor Effects of Ganoderma lucidum (Reishi) in Inflammatory Breast Cancer in In Vivo and In Vitro Models
Source: PLoS One. 2013 Feb 28;8(2):e57431. doi: 10.1371/journal.pone.0057431 (PMC3585368; doi:10.1371/journal.pone.0057431)
Supplement: Table S2 — In vivo expression patterns of PI3K/Akt pathway genes. This table includes all genes that show tendency to be significantly up- or down- regulated with 0.5 mg/ml Reishi at a P value between 0.06 and 0.08 when where compared to vehicle controls. See Table 2 for genes that are significantly regulated and are analyzed at −1.3≥1.3 log2-fold changes. (DOCX) [file pone.0057431.s006.docx]

**Table S2. *In vivo* expression patterns of PI3K/Akt pathway genes.**

| **Gene symbol** | **Complete name** | **Fold change** |
| --- | --- | --- |
| *IRS1* | Insulin receptor substrate 1 | -1.8 |
| *NFKB1* | Nuclear factor of kappa light polypeptide gene enhancer in B-cells 1 | 1.3 |
| *TSC2* | Tuberous sclerosis 2 | -2.0 |

This table include all genes that show tendency to be significantly up- or down- regulated with 0.5mg/ml Reishi at a P value between 0.06 and 0.08 when where compared to vehicle controls. See Table 2 for genes that are significantly regulated and are analyzed at -1.3 ≥ 1.3 log_2_-fold changes.
